# Cyclodextrin-induced release of drug-entrapping liposomes associated with the solation of liposome gels†

**DOI:** 10.1039/d2ra03837d

**Published:** 2022-08-10

**Authors:** Hiromu Yamada, Keita Yamana, Riku Kawasaki, Kazuma Yasuhara, Atsushi Ikeda

**Affiliations:** Applied Chemistry Program, Graduate School of Advanced Science and Engineering, Hiroshima University 1-4-1 Kagamiyama Higashi-Hiroshima 739-8527 Japan; Division of Materials Science, Graduate School of Science and Technology and Center for Digital Green-innovation, Nara Institute of Science and Technology 8916-5 Takayama-cho Ikoma Nara 630-0192 Japan

## Abstract

In this work, we demonstrate that liposome gels in which liposomes are connected by polyethylene glycol terminated by cholesterol groups at both ends can store hydrophilic and hydrophobic drugs in the gel interiors, inner aqueous phases, and lipid membranes. The addition of cyclodextrins (CDxs) as extrinsic stimuli led to the release of drug-entrapping liposomes due to the interactions between CDxs and cholesteryl groups and/or the alkyl chains of lipids. The addition of aqueous solutions of β-CDx, dimethyl-β-CDx, trimethyl-β-CDx, and γ-CDx (final concentration: 7.5 mM) induced the solation of liposome gels and the release of liposomes accompanying the solation. Furthermore, the addition of β-CDx led to the partial release of hydrophilic drugs encapsulated in the liposomes, although the drug release was scarcely observed in other CDxs. In particular, the addition of trimethyl-β-CDx, which has low cytotoxicity, accelerated solation, and cationic liposomes released from the gels were effectively taken up by murine colon cancer (Colon26) cells. Thus, we propose that liposomes released from liposome gels can function as drug carriers.

## Abstract 2

The solation of liposome gels owing to the addition of trimethyl-β-cyclodextrin (TMe-β-CDx) and the uptake of cationic liposomes released from liposome gels by Colon26 cells are demonstrated.

## Graphical abstract

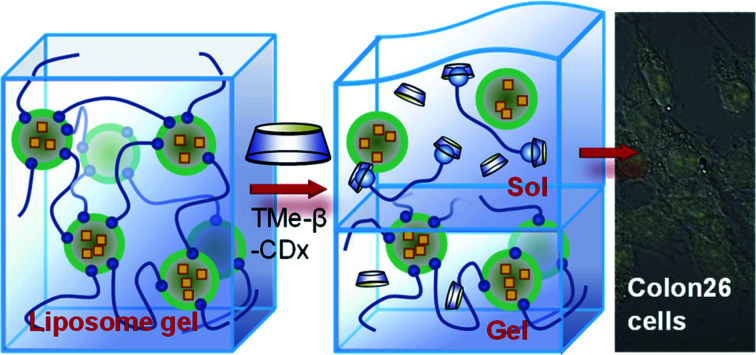

## Introduction

**Fig. 1:**
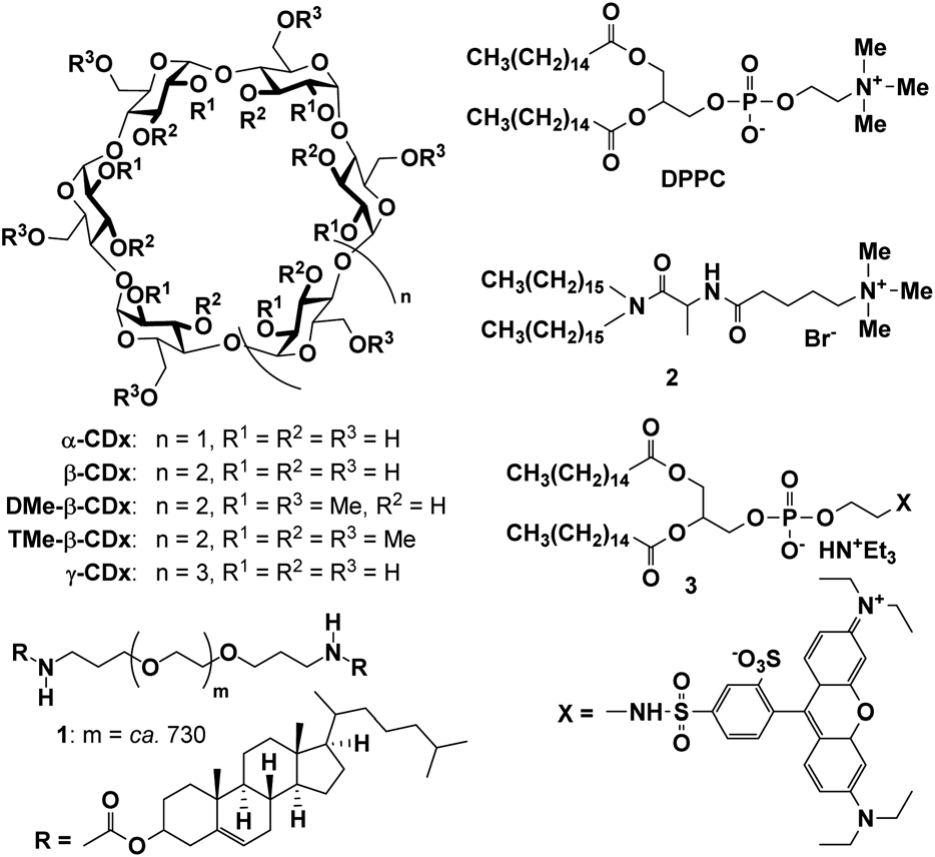
Structures of the compounds used in this study.

**Scheme 1:**
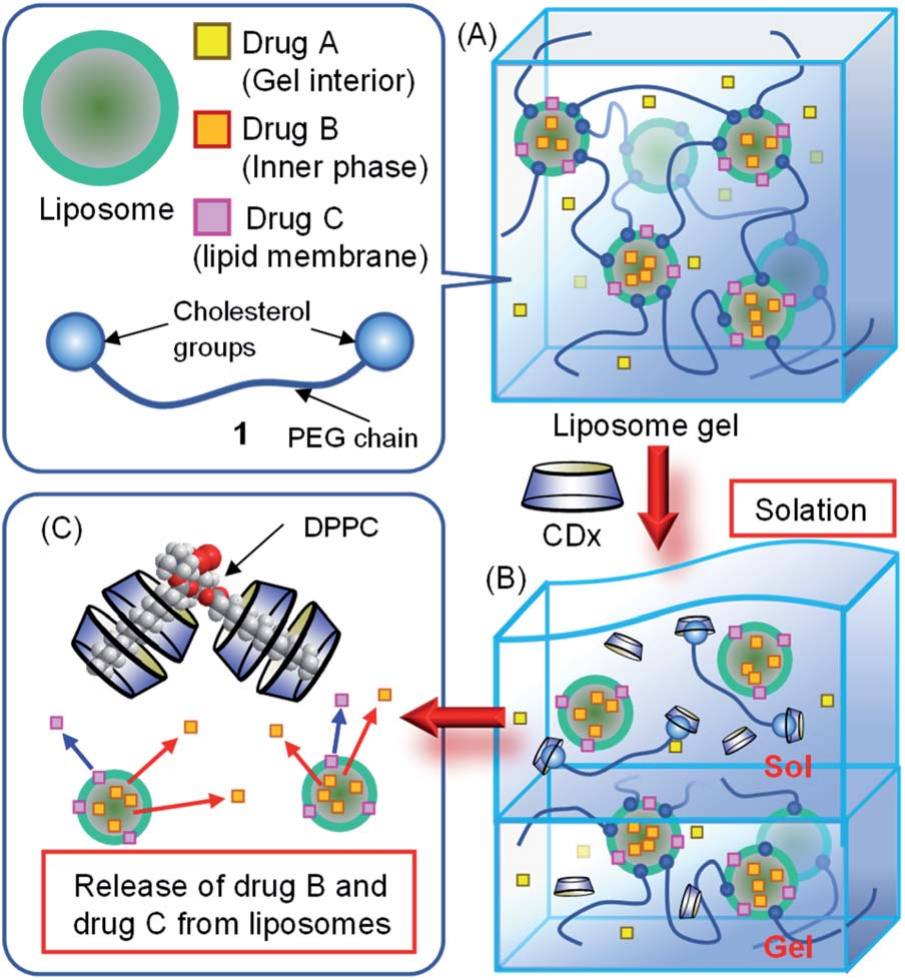
Schematic illustration of (A) liposome gel structure, (B) solation and release of drug A from the gel interior, and (C) release of drug B from the inner aqueous phase of liposomes and drug C from the lipid membrane of liposomes by the addition of CDxs.

Liposomes have attracted immense interest as highly efficient drug carriers.^1–5^ Liposomes consisting of phospholipids possess high biocompatibility and passive tumor targeting *via* the enhanced penetration and retention effect.^6,7^ Furthermore, hydrophilic and hydrophobic drugs can be incorporated into the inner aqueous phase and lipid membranes, respectively.^2,3,8–12^ Injectable gelation^13–15^ of aqueous solutions containing liposomes, which incorporate hydrophilic or hydrophobic drugs, is crucial for the long-term retention of drugs in liposomes, their accumulation at disease sites, and the controlled release of drugs in the body. Taguchi *et al.* reported that liposomes connected by polyethylene glycol derivatives with two cholesterol terminals (Fig. 1, 1) form supramolecular hydrogels, called liposome gels (Scheme 1).^16,17^ We employed cyclodextrins (CDxs) as an external trigger to introduce a stimuli-responsive feature for obtaining liposome gels. CDxs can interact with cholesterol and the alkyl chains of lipids.^18–25^ These interactions can pull out cholesterol from cell membranes^18,19^ or collapse cell membranes.^20–25^ Furthermore, free CDxs can be released from pseudo-rotaxane or rotaxane *via* external stimuli such as light and pH change.^26,27^ Depending on the affinity of CDxs to lipids, the cytotoxicities of CDxs and their derivatives were reported to be in the following order: heptakis(2,6-di-*O*-methyl)-β-cyclodextrin (DMe-β-CDx) ≫ β-CDx > heptakis(2,3,6-tri-*O*-methyl)-β-cyclodextrin (TMe-β-CDx) ≈ α-CDx > γ-CDx. Correspondingly, CDxs have low toxicity, except for DMe-β-CDx.^28–30^ It is predicted that the addition of CDxs can elute liposomes from the liposome gel, triggering the release of substances encapsulated in liposomes (Scheme 1). In this work, we investigated the solation of liposome gels accompanied by the release of substances from the inner aqueous phases of liposomes by the addition of CDxs. Further, we confirmed the intracellular uptake of cationic liposomes by murine colon cancer (Colon26) cells after the solation of the liposome gels.

## Results and discussion

### Solation of liposome gels by addition of cyclodextrins

**Fig. 2:**
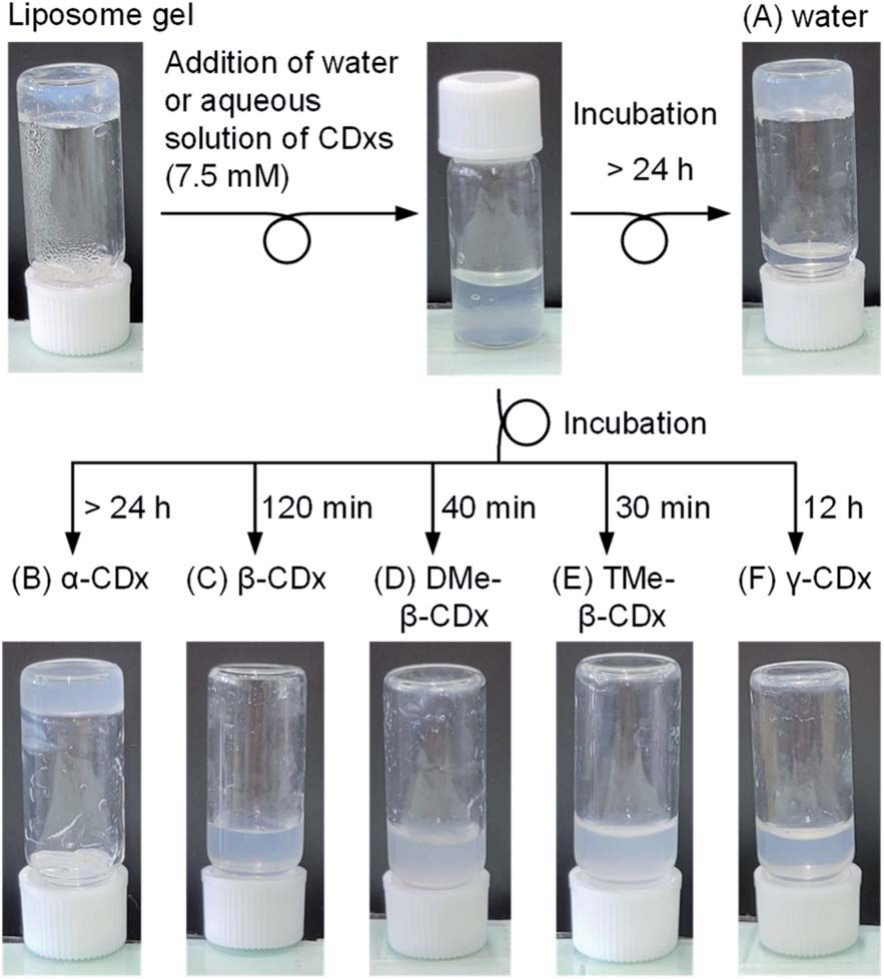
Photographic images of the liposome gel after the addition of (A) water and aqueous solutions of (B) α-CDx, (C) β-CDx, (D) DMe-β-CDx, (E) TMe-β-CDx, and (F) γ-CDx ([DPPC] = 2.5 mM, 1: 1.5% (w/v), [CDx] = 0 or 7.5 mM).

**Fig. 3:**
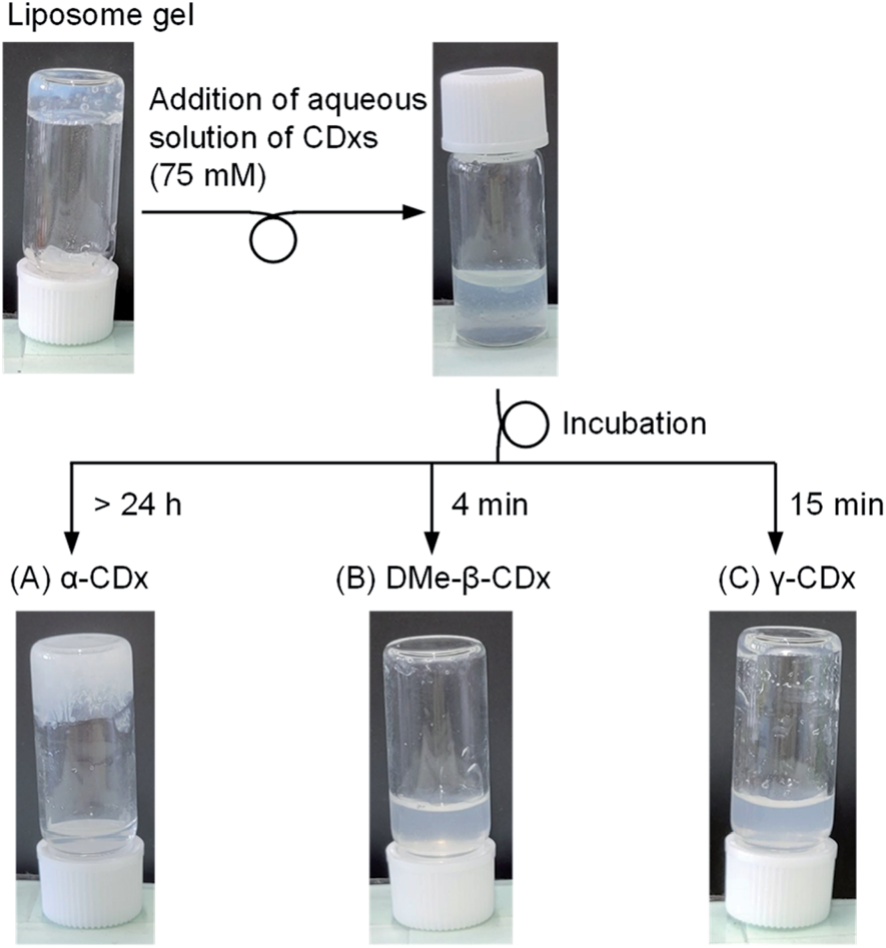
Photographic images of theliposome gel after the addition of aqueous solutions of (A) α-CDx, (B) DMe-β-CDx, and (C) γ-CDx at high concentrations ([DPPC] = 2.5 mM, 1: 1.5% (w/v), [CDx] = 75 mM).

To investigate the effect of CDxs addition on sol–gel states, aqueous solutions of various CDxs were added to the liposome gel (Fig. 2). The concentration of CDxs was adopted to be 15 mM (aqueous solutions) because all CDxs, including β-CDx which had lowest solubility in water (15.9 mM), were soluble in water in the same concentration. The final concentration of CDxs became 7.5 mM upon mixing an equal volume of the liposome gel ([DPPC] = 5.0 mM, 1: 3.0% (w/v), 600 μL) and an aqueous solution of CDxs (600 μL). To obtain stable liposome gel, we employed [DPPC] = 5.0 mM, 1: 3.0% (w/v).^17^ After the incubation at room temperature for 24 h, the disappearance of the lump of the liposome gel was observed. We propose two mechanisms for the solation of the liposome gels by the addition of CDxs: (i) CDx pulls out cholesterols from liposomes by interacting with the cholesterol moiety, and (ii) CDx collapses liposomes by interacting with 1,2-dipalmitory-*sn-glycero*-3-phosphocholine (DPPC). After the addition of water or an aqueous solution of α-CDx (7.5 mM), there was no change in the liposome gel (Fig. 2A and B). Consequently, α-CDx could not induce liposome gel solation by either mechanism (i) or (ii). This is expected for mechanism (i) since α-CDx scarcely interacts with cholesterol because α-CDx has an extremely small cavity so as not to be able to include the steroid structure.^31,32^ Regarding mechanism (ii), although it is known that α-CDx can interact with the alkyl chains of lipids and collapse liposomes,^18–25^ their interactions increase turbidity without solation (Fig. 2B). The enhanced light scattering was caused by the increase in liposome size due to the interaction between α-CDx and DPPC (Table S1†), but the concentration of α-CDx was insufficient to collapse the liposomes. In contrast, by incubation after the addition of the aqueous solutions of β-CDx, DMe-β-CDx, TMe-β-CDx, and γ-CDx, the complete solation of the liposome gel was observed ([CDx] = 7.5 mM) (Fig. 2C–F). The complete solation took 30–120 min for β-CDx, DMe-β-CDx, and TMe-β-CDx, but 12 h for γ-CDx. γ-CDx slowly extracts 1 because it shows the second weakest interaction with cholesterol among the CDxs examined in this study (Fig. 3C).^31^ Whether these solations were obtained by mechanism (i) or (ii) were investigated by performing the following experiments.

Additional effects caused by higher concentrations of CDxs were examined for α-CDx, DMe-β-CDx, and γ-CDx because these CDxs can be readily dissolved (over 150 mM) in water. By increasing the concentration of α-CDx (final concentration: 75 mM), the turbidity of the liposome gel was enhanced, but the gel state remained (Fig. 3A). The result indicates that α-CDx, even at a high concentration, could not induce solation by the collapse of liposomes (mechanism (ii)). In contrast, the solation was accelerated to 15 min using a 150 mM (final concentration: 75 mM) aqueous solution of γ-CDx (Fig. 3C). The treatment using DMe-β-CDx (75 mM) similarly induced faster solation than that by 15 mM DMe-β-CDx (Fig. 3B). Notably, the experiments with high concentrations of β-CDx and TMe-β-CDx could not be conducted because of their low solubility in water.^33,34^ Thus, the following experiments were performed using CDxs, except for α-CDx, as external stimuli to introduce solation.

### Collapse of liposomes by addition of cyclodextrins as confirmed by calcein release

**Fig. 4:**
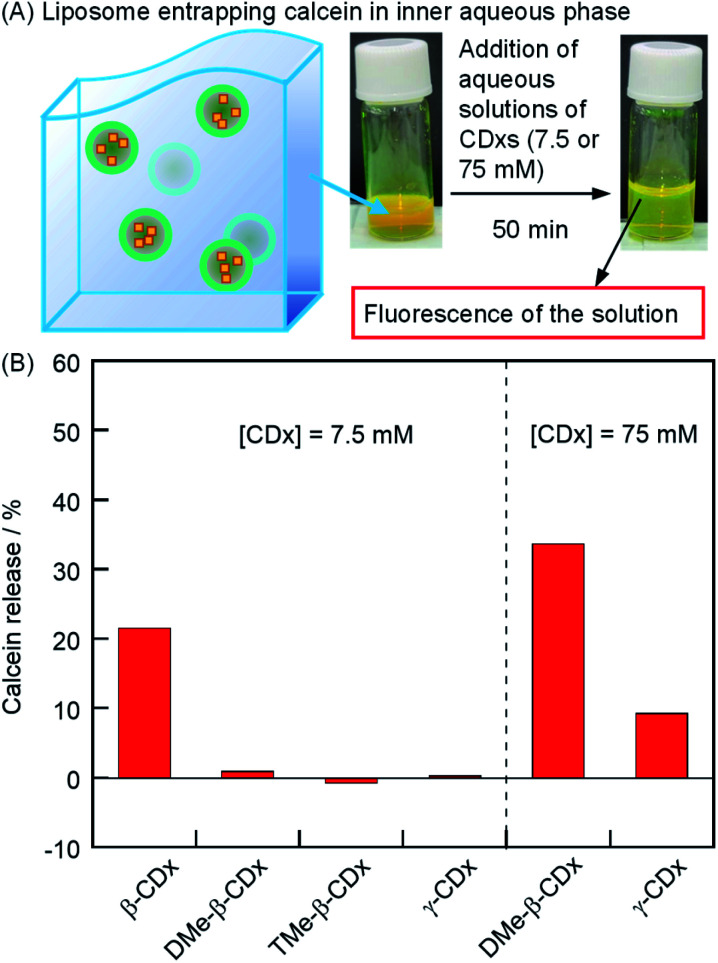
(A) Photographic images of liposomes entrapping calcein in the inner aqueous phase after the addition of aqueous solutions of CDx. (B) Percentage of calcein release by the addition of CDx in the absence of 1 after incubation for 50 min ([DPPC] = 2.5 mM, [CDx] = 7.5 or 75 mM).

To investigate whether the collapse of liposomes (mechanism (ii)) occurs by the addition of CDxs, we used liposomes that encapsulated calcein in the inner aqueous phase.^35,36^ Since calcein was encapsulated at a sufficiently high concentration to induce self-quenching, the release of calcein from the liposomes in the liposome gel was monitored by the recovery of the fluorescence intensity upon the dilution of calcein in the outer aqueous phase (Fig. 4). The release percentage of calcein from the inner aqueous phase of the vesicle to the outer phase (drug B in Scheme 1) was calculated by

1 calcein release (%) = (*F* − *F*_0_)/(*F*_T_ − *F*_0_),

where *F*_0_, *F*, and *F*_T_ are the calcein fluorescence intensities of the DPPC liposome encapsulating calcein, the liposome gel after the addition of each CDx, and the liposome gel (100 μL) after the addition of 222 mM Triton X-100 (10 μL, final concentration: 20 mM), respectively (Fig. S1†). Fluorescence intensities were corrected for dilution. In the absence of 1, by the addition of DMe-β-CDx, TMe-β-CDx, or γ-CDx ([CDx] = 7.5 mM), the calcein was scarcely released in 50 min (Fig. 4B). In contrast, 22% of calcein was released from liposomes by the addition of β-CDx (Fig. 4B). At present, we could not determine whether the solation by β-CDx was triggered by mechanism (i) or (ii). When a larger amount of DMe-β-CDx or γ-CDx was added (75 mM), the release of calcein increased dramatically (34 and 9%, respectively). The plausible mechanisms for calcein release by β-CDx, DMe-β-CDx, and γ-CDx are discussed in the section on the morphology of liposomes.

### Determination of calcein release to the outer aqueous phase of liposomes

**Fig. 5:**
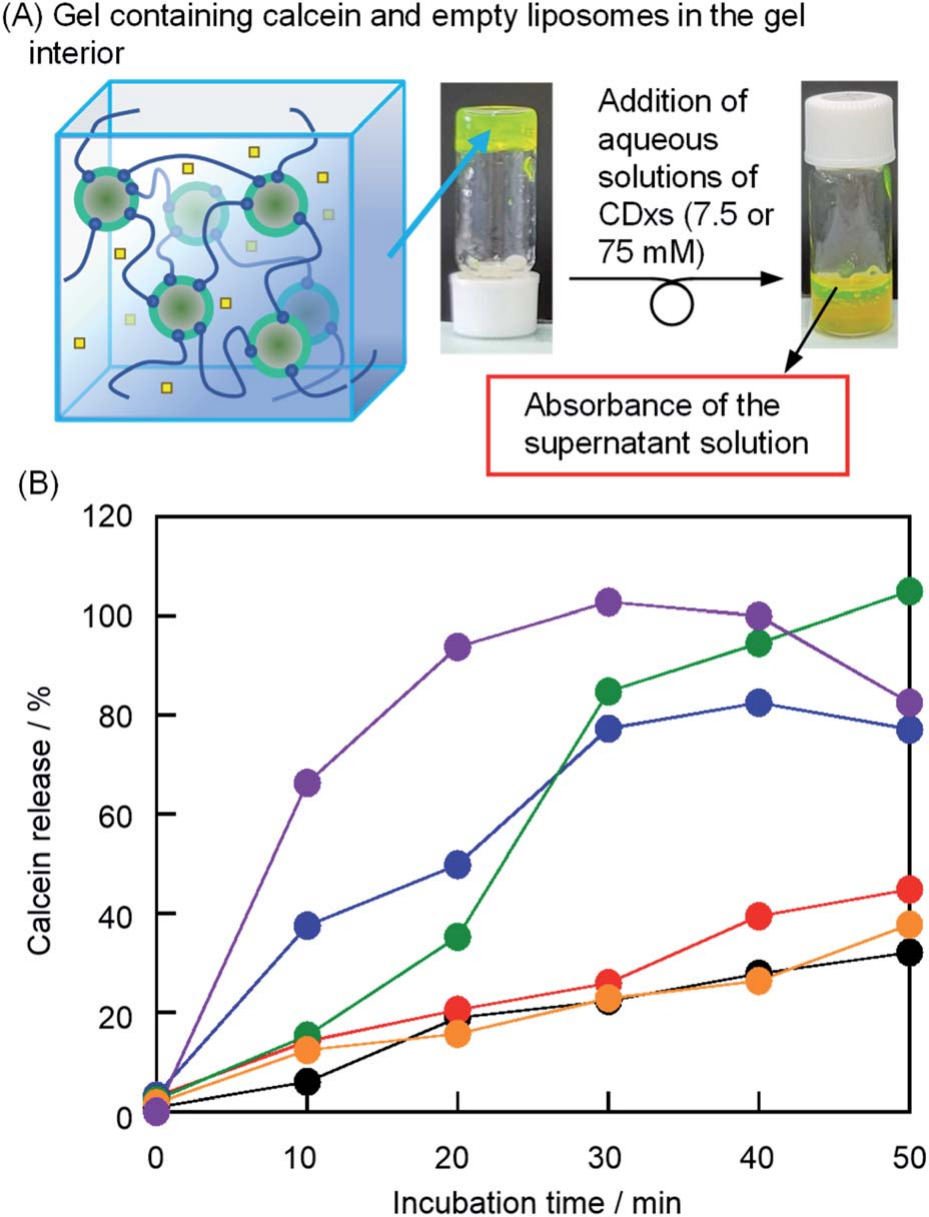
(A) Photographic images of the liposome gel containing calcein and empty liposomes in the gel interior after the addition of CDx. (B) Incubation time dependence of the absorbance at *λ*_max_ of calcein in the bulk solution with the addition of water (black), 15 mM aqueous solutions of β-CDx (red), DMe-β-CDx (blue), TMe-β-CDx (green), and γ-CDx (orange), and 150 mM aqueous solution of γ-CDx (purple) ([DPPC] = 2.5 mM, 1: 1.5% (w/v), [CDx] = 0, 7.5 or 75 mM).

The release of hydrophilic substrates (drug A in Scheme 1) from the outer aqueous phase of the liposomes was monitored by the fluorescence intensity of calcein transferred from the gel interior to bulk water (Fig. 5). By the addition of water without CDxs, the calcein release gradually increased to approximately 30% in 60 min (Fig. 5B). Because the addition of water did not result in solation, the release of calcein from the gel interior was caused by diffusion. Therefore, when the aqueous solutions of CDxs were added, the calcein release exceeding that by the addition of water was accompanied by the solation of liposome gels. Since the absorbance of calcein decreased due to the formation of β-CDx–calcein and TMe-β-CDx–calcein complexes (Fig. S2†), the values of *F*_0_, *F*, and *F*_T_ were corrected by the absorbances of these complexes. After the addition of the aqueous solutions of β-CDx, DMe-β-CDx, TMe-β-CDx, and γ-CDx (15 mM), the calcein releases, *i.e*., the solation rates increased in the order of DMe-β-CDx > TMe-β-CDx ≫ β-CDx > γ-CDx ≈ water (Fig. 5B and S3†). In contrast, after the addition of the aqueous solution of γ-CDx (150 mM), the solation of liposome gels occurred quickly (Fig. 5B). These results agreed with the observations of solation, shown in Fig. 2.

### Release of calcein-encapsulated in the inner aqueous phase of liposomes by the addition of CDxs

**Fig. 6:**
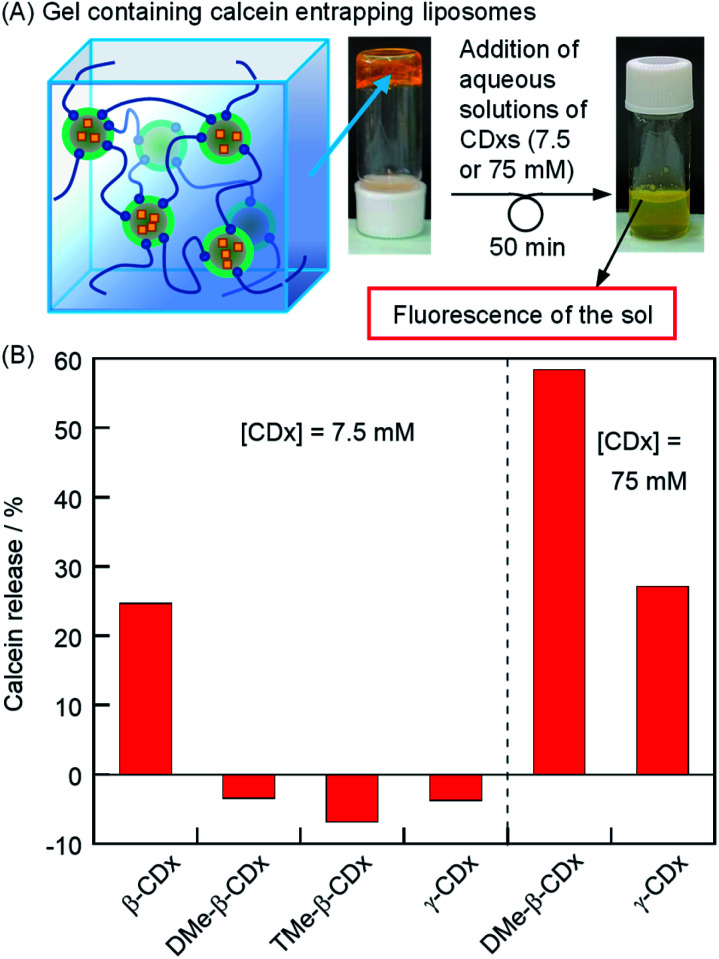
(A) Photographic images of the gel containing calcein entrapping liposomes after the addition of aqueous solutions of CDx. (B) Percentage of calcein release with the addition of CDx in the presence of 1 after incubation for 50 min ([DPPC] = 2.5 mM, 1: 1.5%(w/v) [CDx] = 7.5 or 75 mM).

To determine the integrity of liposomes in a gel, the release of vesicle-encapsulated calcein was observed by fluorescence (Fig. 6A). Liposome gels were prepared using liposomes that encapsulated calcein at a self-quenching concentration (50 mM) in physiological saline. The release percentages of calcein from the inner aqueous phase of the vesicle to the outer phase (drug B in Scheme 1) were also calculated by eqn (1) (Fig. S4†), where *F*_T_ is the calcein fluorescence intensity of the liposome gel after the addition of 222 mM Triton X-100 (10 μL, final concentration: 20 mM) in the absence of 1 and CDxs because the presence of 1 and CDxs increased the turbidity of the sample, which remarkably decreased its fluorescence intensity due to scattering. These values were corrected for dilution, for example, by the addition of the aqueous solutions of CDxs and Triton X-100. The results of calcein release are shown in Fig. 6B. The addition of β-CDx aqueous solution led to a calcein release of 25% ([β-CDx] = 7.5 mM). The value was similar to that in the absence of 1 (Fig. 4B), indicating that the influence of 1 was exceedingly small toward the release of calcein. In contrast, the percentages of the calcein release became slightly negative by the addition of DMe-β-CDx, TMe-β-CDx, and γ-CDx as shown in Fig. 6B, because of the reduction of fluorescence intensity due to the slight change in the scattering of liposomes (the inference is supported by the increase in the hydrodynamic diameters of DPPC liposomes after the addition of CDxs; Table 1). Therefore, these results indicate that the addition of these CDxs scarcely induced the release of calcein. Despite the extremely high affinity of DMe-β-CDx to lipids, the percentage of calcein release was −3% by the addition of a 15 mM aqueous solution of DMe-β-CDx. Because three or four DMe-β-CDx molecules need to interact with the alkyl chains of one lipid,^22–24^ the concentration of DMe-β-CDx was too small to collapse the liposomes, as well as α-CDx. Furthermore, similar percentages of calcein release were observed in the absence and presence of 1 (1 and −3%, respectively), indicating that the calcein release was scarcely influenced by 1 (Fig. 6B). In contrast, 58 and 27% of calcein were released from liposomes by DMe-β-CDx and γ-CDx (75 mM), respectively (Fig. 6B). The percentages of calcein release increased significantly compared with that in the absence of 1 (Fig. 4B: 34 and 9%, respectively). The reason is not clear at present.

**Table 1:** Hydrodynamic diameters (*D*_hy_) of sols^a^ after the addition of CDx determined by dynamic light scattering at 25 °C

CDx	1/% (w/v)	[CDx]/mM	[DPPC]/mM	*D*_hy_ b/nm	PDI^c^
—	0	0	2.5	107.1 ± 0.9	0.07
β-CDx	1.5	7.5	2.5	174.9 ± 3.2	0.17
DMe-β-CDx	1.5	7.5	2.5	154.2 ± 1.8	0.22
TMe-β-CDx	1.5	7.5	2.5	157.4 ± 1.5	0.18
γ-CDx	1.5	7.5	2.5	114.8 ± 57.5	0.92

a Sol solutions were diluted to 1/10 by water. The final concentrations of CDx and DPPC were 0.75 and 0.25 mM, respectively.

b Each experiment was performed in triplicates.

c PDI: polydispersity index.

### Release of rhodamine B by the addition of TMe-β-CDx

**Fig. 7:**
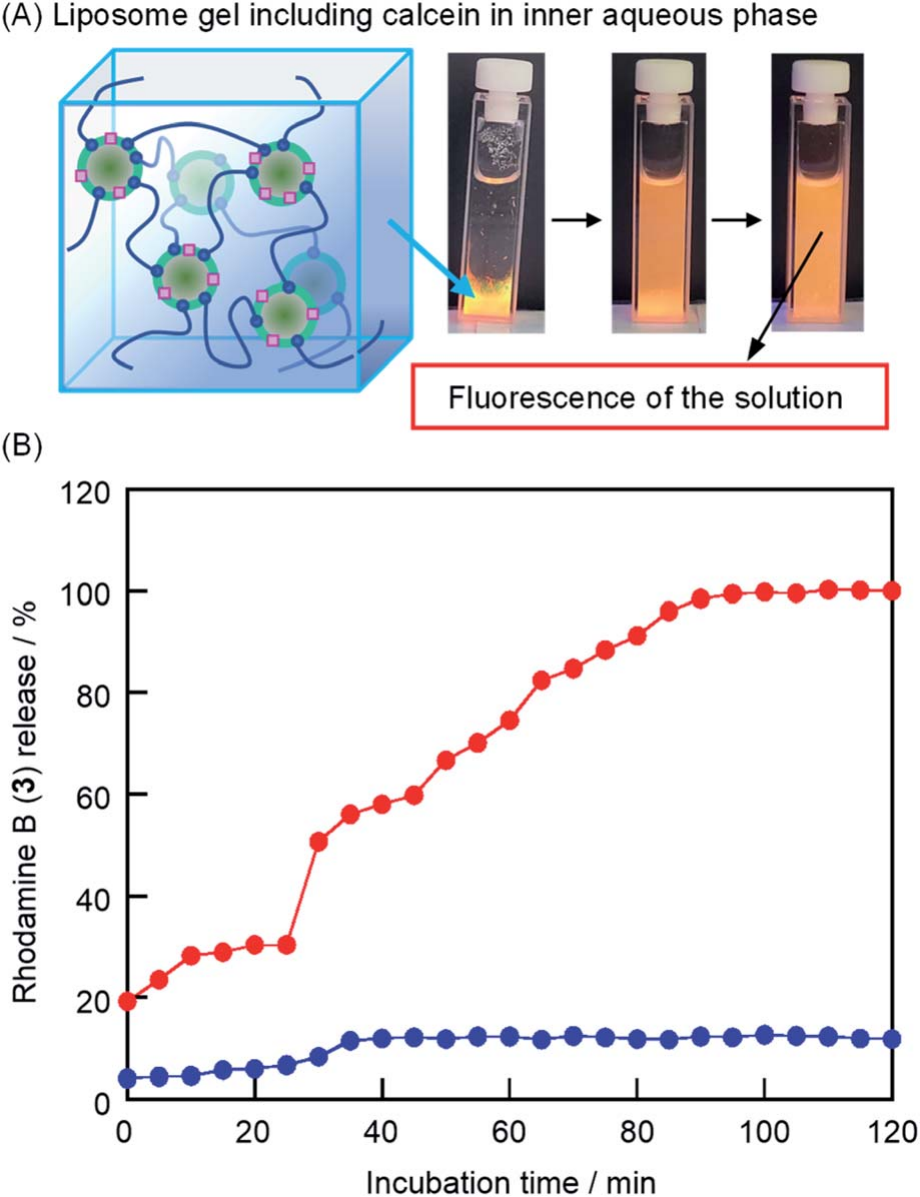
(A) Photographic images of the liposome gel incorporated in the lipid membranes of 3 after the addition of the aqueous solutions of TMe-β-CDx. (B) Incubation time-dependence of the absorbance at *λ*_max_ of 3 in the bulk solution with the addition of TMe-β-CDx (red) and saline (blue) ([DPPC] = 2.5 mM, 1: 1.5%(w/v), [3] = 25 μM, [3]/[DPPC] = 1.0 mol%, red: [TMe-β-CDx] = 7.5 mM, blue: [TMe-β-CDx] = 0 mM).

We observed the release of liposomes from the liposome gel based on the fluorescence intensity in the bulk water using a lipid membrane incorporating rhodamine B 1,2-dihexadecanoyl-*sn-glycero*-3-phosphoethanolamine (3) as drug C (Scheme 1, Fig. 7A, [3]/[DPPC] = 1.0 mol%). In this experiment, we employed TMe-β-CDx because of its low cytotoxicity, low liposome collapse ratio, and fast liposome gel solation. The incubation time for the complete solation in this experiment (90 min) was longer than that of the visible observation (Fig. 7B and 2E). The reason was that, in this measurement, the solutions were kept back in UV cells without sampling. As shown in Fig. S5A,† we observed the increase in not only the absorbance of 3 at 573 nm but also the scattering of light by liposomes in the 300–800 nm region (Fig. S5A†). Consequently, the lipid membrane incorporated in 3 was released from the liposome gel by TMe-β-CDx. In contrast, the spontaneous release from liposomes was scarcely observed in the absence of CDxs (Fig. 7B and S5B†).

### Morphology of liposomes released from liposome gels

**Fig. 8:**
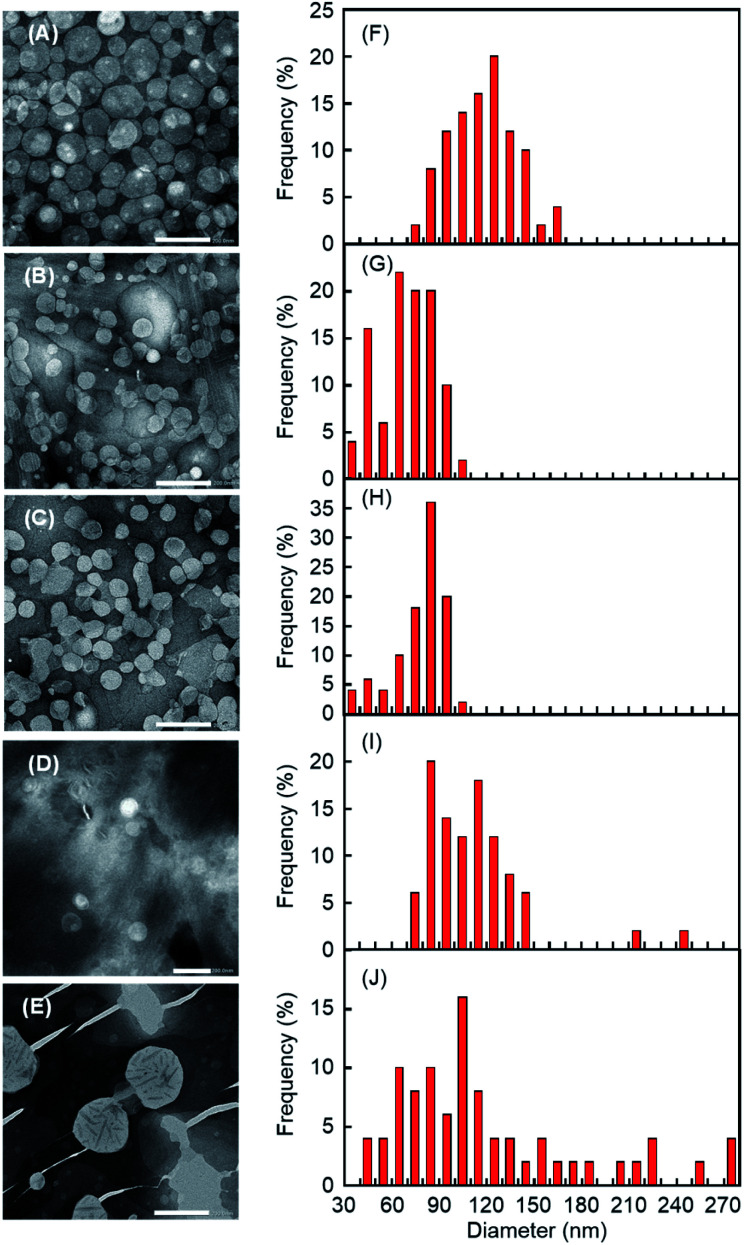
(A–E) TEM images of the sol solution obtained from liposome gel after the addition of 15 mM aqueous solutions (600 μL) of (A) β-CDx, (B) DMe-β-CDx, (C) TMe-β-CDx and (D) γ-CDx and 150 mM aqueous solution of (E) DMe-β-CDx ([DPPC] = 2.5 mM, 1: 1.5% (w/v) [CDx] = 7.5 or 75 mM). The scale bars correspond to 200 nm. (F–J) Histograms of nanoparticle diameters determined from TEM images after the addition of 15 mM aqueous solutions of (F) β-CDx, (G) DMe-β-CDx, (H) TMe-β-CDx and (I) γ-CDx and 150 mM aqueous solution of (J) DMe-β-CDx. Each histogram was calculated based on 50 particles randomly selected from TEM images.

After the addition of CDxs (except α-CDx), we ascertained the morphology of DPPC liposomes in the sol. Transmission electron microscopy (TEM) observations for the addition of aqueous solutions of β-CDx, DMe-β-CDx, TMe-β-CDx, and γ-CDx (final concentration: 15 mM) in the liposome gels revealed that lipid vesicles remained for all cases of CDxs (Fig. 8A–D and S6A–D†). The sizes of liposomes obtained by the addition of aqueous solutions of β-CDx were larger than those obtained by the addition of other CDxs (Fig. 8F–I). A fusion among liposomes by β-CDx led to not only the size increase of liposomes but also the release of liposome-encapsulated calcein, as shown in Fig. 4B and 6B. In contrast, by the addition of γ-CDx, liposomes with similar size to those of DMe-β-CDx and TMe-β-CDx were observed, but the number of liposomes decreased (Fig. 8D). It is likely that the self-aggregation of 1^16,17^ after the solation influenced the TEM image because of the weak interaction between 1 and γ-CDx. The addition of DMe-β-CDx at a high concentration (75 mM) led to the collapse of liposomes (Fig. 8E, J and S6E†). This result indicates that the collapse of liposomes led to high calcein release values (58%) from the inner aqueous solution in the absence and presence of 1, respectively (Fig. 4B and 6B).

Furthermore, we measured the hydrodynamic diameters (*D*_hy_) of DPPC liposomes by a dynamic light scattering (DLS) spectrophotometer (Table 1 and Fig. S7†). The *D*_hy_ value obtained by the addition of β-CDx was larger than those of DMe-β-CDx and TMe-β-CDx, which agreed with the liposome sizes in TEM images (Fig. 8F–H). The result indicates the fusion among liposomes with the addition of β-CDx. After the addition of 15 mM aqueous solutions of DMe-β-CDx and TMe-β-CDx, the *D*_hy_ values of DPPC liposomes were approximately constant both in the absence and presence of 1 (Table 1, 143–157 nm). In these systems, because of the low percentages of calcein release by the addition of these CDxs (Fig. 6B), the slight increase in *D*_hy_ values was expected due to swelling. In contrast, the *D*_hy_ value was scarcely changed after the addition of γ-CDx (115 nm), but a large PDI value was obtained. The result also suggests that the self-aggregation of 1^16,17^ was formed because γ-CDx scarcely interact with cholesterol moieties of 1.

### Intracellular uptake of liposomes released from liposome gels by Colon26 cells

**Fig. 9:**
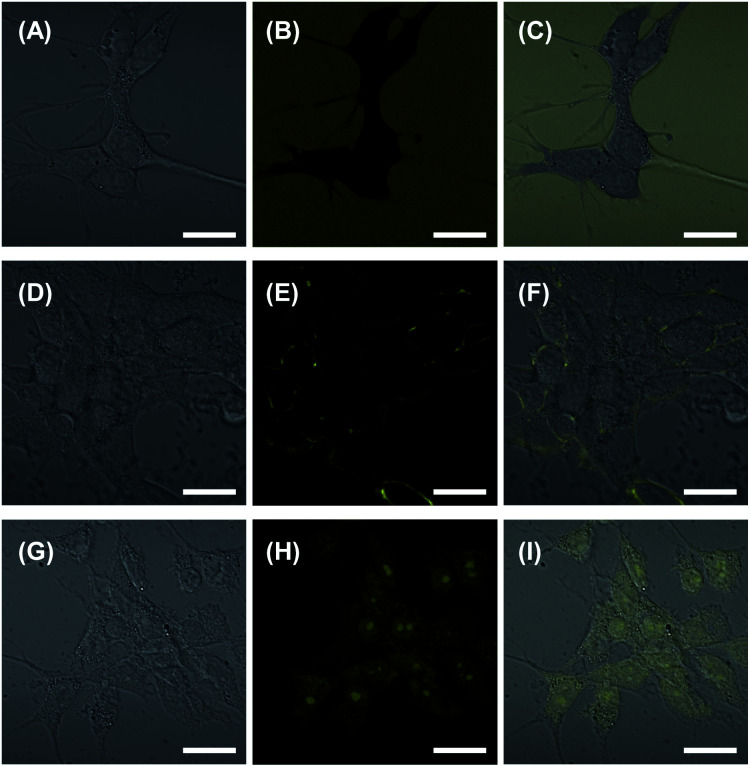
Differential interference contrast (A, D, and G), fluorescence (B, E, and H) and overlay images (merge) (C, F, and I) of Colon26 cells treated with an aqueous solution of calcein (A–C) and liposome including calcein in the inner aqueous phase without (D–F) and with (G–I) TMe-β-CDx for 12 h at 37 °C. The scale bar represents 10 μm.

Before the experiments of the intracellular uptake, the cytotoxicity of the constituent compounds in this system was investigated. It was already known that DPPC and TMe-β-CDx have low cytotoxicity.^28–30,37,38^ Furthermore, a cytotoxicity of 1 was tested in the presence of TMe-β-CDx because 1 forms gel by itself in the absence of liposomes.^16,17^ Compound 1 showed a low cytotoxicity toward murine colon cancer (Colon26) cells until the concentration of 0.075 wt% after 24 h incubation (Fig. S8†). The intracellular uptake of the liposomes upon the addition of TMe-β-CDx by murine colon cancer (Colon26) cells were observed by confocal laser scanning microscopy (CLSM) at the emission wavelengths of calcein (Fig. 9). We prepared the liposomes containing 10 mol% cationic lipid 2 toward DPPC to aid the intracellular uptake by Colon26 cells with an anionic surface.^35–37^ Dilute calcein was entrapped in the inner aqueous phase for fluorescence observation. As shown in Fig. 9C, while the fluorescence was scarcely observed in Colon26 cells by the addition of an aqueous solution of calcein, it was observed on the glass culture plate (Fig. 9B and C). Furthermore, in the absence of TMe-β-CDx, the liposome entrapping calcein in the inner aqueous phase was scarcely incorporated in Colon26 cells (Fig. 9F). In contrast, in the presence of TMe-β-CDx, we observed a fluorescence signal in the Colon26 cells (Fig. 9I). The results indicate that the cationic liposomes entrapping calcein after the release by TMe-β-CDx were successfully delivered to and incorporated by Colon26 cells, indicating that liposomes released from the liposome gels can function as drug carriers.

## Conclusions

The addition of α-CDx did not induce the solation of liposome gels because α-CDx cannot form a complex with the cholesterol moiety of 1. In contrast, the addition of 7.5 mM aqueous solutions of DMe-β-CDx, TMe-β-CDx, and γ-CDx induced the solation of liposome gels with the release of liposomes. Furthermore, the release of liposomes by DMe-β-CDx and TMe-β-CDx was faster than that by γ-CDx. At this time, the solation led to the release of hydrophilic guest molecules from the gel interior but not from the inner aqueous phase of liposome gels. Consequently, in a local site such as a subcutaneous injection, the addition of DMe-β-CDx, TMe-β-CDx, and γ-CDx allows the controllable release of liposomal drugs from liposome gels. The addition of a 7.5 mM aqueous solution of β-CDx and 75 mM aqueous solutions of DMe-β-CDx and γ-CDx induced not only the solation of liposome gels but also the release of the hydrophilic drug from the inner aqueous phase of liposomes. Furthermore, the hydrophobic drugs incorporated in the lipid membrane were released along with liposomes from liposome gels. It is conceivable that the release of various drugs from the inner and outer aqueous phases and lipid membranes of liposome gels can be controlled by the selection of the types and concentrations of CDxs.

## Experimental

### Materials

1,2-Dipalmitoyl-*sn-glycero*-3-phosphocholine (DPPC) was purchased from NOF Corp. (Tokyo, Japan). α-CDx, β-CDx, TMe-β-CDx, and γ-CDx were obtained from Wako Pure Chemical Industries Ltd (Tokyo, Japan). DMe-β-CDx was purchased from Sigma-Aldrich Chemical Co., Inc. (St Louis, MO, USA). Calcein disodium salt was purchased from Cosmo Bio Co. Ltd (Tokyo, Japan). Compound 1 was prepared as described in a previous study.^16^

### Preparation of liposomes

A chloroform solution of DPPC (300 μL, 50 mM), mixture of DPPC (180 μL, 50 mM) and 2 (100 μL, 10 mM), or mixture of DPPC (200 μL, 50 mM) and 3 (100 μL, 1.0 mM) was air-dried. The thin film was added saline (1.0 mL) and heated at 55 °C. The mixture was shaken on a vortex mixer for 5 min. To change from multilamellar to unilamellar vesicles and obtain a narrow size distribution, the solution was subjected to freeze–thaw cycles (8 times) and extruded 31 times (LiposoFast-Basic; Avestin Inc., Ottawa, Canada) with two stacked polycarbonate membranes (pore size: 50 nm). The resulting solution was diluted with water to a final concentration of 15.0 mM lipids.

Calcein-encapsulated DPPC liposome was obtained using a chloroform solution of DPPC (200 μL, 50 mM) or a mixture of DPPC (180 μL, 50 mM) and 2 (100 μL, 10 mM) and saline containing 50 mM calcein (1.0 mL) by the same procedure mentioned above. To remove calcein in the outer aqueous phase, calcein-encapsulated DPPC liposome was purified by gel column chromatography (Sephacryl S-400) using saline as the eluent.

### Preparation of liposome gels

According to a previous study,^16^ three types of liposome gels were prepared by mixing (i) 1 (18 mg), DPPC liposome (133 μL, 15 mM), calcein-encapsulated DPPC liposome (250 μL), and saline (217 μL), (ii) 1 (18 mg), DPPC liposome (133.3 μL, 15 mM), calcein-encapsulated DPPC liposome (250 μL) and pure water (216.7 μL), or (iii) 1 (18 mg), DPPC liposome (300 μL, 10 mM), and aqueous solution of calcein (300 μL, 50 mM) at 55 °C ([DPPC] = 5.0 mM, 1; 3.0% (w/v), 600 μL). The resulting gels were obtained in the absence and presence of calcein inside and outside of DPPC liposomes, respectively.

### Addition of CDxs and Triton X-100 in liposome gels

The aqueous solutions (600 μL) of the DPPC liposome gel (5.0 mM), calcein-encapsulated DPPC liposome gel, and DPPC liposome gel in the presence of calcein were added to the aqueous solutions (600 μL) of CDxs (15 or 150 mM). The samples were incubated at ambient temperature.

### UV-vis and fluorescence spectroscopy

UV-vis absorption spectra were recorded using a UV-3600PC spectrophotometer (Shimadzu Corp., Kyoto, Japan). All experiments were performed at 25 °C using cell path length of 1 cm. Fluorescence spectra were measured using an F-2700 fluorescence spectrophotometer (Hitachi, Tokyo, Japan). An excitation wavelength of 475 nm was used for calcein along with emission wavelengths in the range of 485–650 nm.

### DLS measurements

The hydrodynamic diameters of DPPC liposomes and liposome gels after the addition of CDxs were measured at 25 °C, using an electrophoretic light scattering instrument with a laser Doppler system (Zetasizer Nano ZS, Malvern Instruments Ltd, Malvern, UK). The sol solutions were diluted to 1/10 by water. The instrument consisted of a He/Ne laser operating at 633 nm and 10 mW. The DTS Nano version 5.00 software was used (Malvern Instruments Ltd, Malvern, UK).

### TEM

The TEM of the released liposomes from liposome gels (600 μL) after the addition of 15 mM aqueous solutions (600 μL) of β-CDx, DMe-β-CDx, TMe-β-CDx, and γ-CDx was performed using a negative staining technique with ammonium molybdate. Sample droplets (2–3 μL) were placed on collodion supported on a TEM grid; then, the liquid was absorbed using filter paper. A drop of aqueous ammonium molybdate solution (1%) was placed on the grid and incubated at room temperature for 1 min. The solution was absorbed using filter paper. Composite morphology was then characterized using a transmission electron microscope (JEM-2010, JEOL Ltd, Tokyo, Japan).

### Cytotoxicity of 1 toward Colon 26

Murine colon cancer (Colon26) cells were seeded on 48 well plate (1.71 × 10^4^ cells) and incubated 24 h. The cells were exposed to 1 which was complexed and solubilized with TMe-β-CDx at an optional concentration (0.0015–0.15 wt% 1 to cell culture medium). After an additional 24 h, cell counting kit-8 (CCK8) solution was added to each sample and the absorbance at 450 nm was measured using a microplate reader (MPR-A100, AS ONE, Tokyo, Japan) to quantify cell viability.

### Cellular uptake study

Colon26 cells were co-incubated with an aqueous solution of calcein or liposome gel in the absence and presence of TMe-β-CDx for 24 h at 37 °C. After incubation, the phase contrast and fluorescence images of Colon26 cells were obtained using CLSM (LSM700, Carl Zeiss, Germany) at an excitation wavelengths of 488 nm.

## Conflicts of interest

There are no conflicts to declare.

## Supplementary Material

RA-012-D2RA03837D-s001

## References

[cit1] Liposomes: A Practical Approach, ed. V. P. Torchilin and W. Weissig, 2nd edn, Oxford University Press, Oxford, 2003

[cit2] Allen T. M., Cullis P. R. (2013). Adv. Drug Delivery Rev..

[cit3] Eloy J. O., de Souza M. C., Petrilli R., Barcellos J. P. A., Lee R. J., Marchetti J. M. (2014). Colloids Surf., B.

[cit4] Hussain A., Singh S., Sharma D., Webster T. J., Shafaat K., Faruk A. (2017). Int. J. Nanomed..

[cit5] Olusanya T. O. B., Ahmad R. R. H., Ibegbu D. M., Smith J. R., Elkordy A. A. (2018). Molecules.

[cit6] Matsumura Y., Maeda H. (1986). Cancer Res..

[cit7] Maeda H., Seymour L., Miyamoto Y. (1992). Bioconjugate Chem..

[cit8] Ikeda A., Sato T., Kitamura K., Nishiguchi K., Sasaki Y., Kikuchi J., Ogawa T., Yogo K., Takeya T. (2005). Org. Biomol. Chem..

[cit9] Ikeda A., Doi Y., Hashizume M., Kikuchi J., Konishi T. (2007). J. Am. Chem. Soc..

[cit10] Ikeda A., Doi Y., Nishiguchi K., Kitamura K., Hashizume M., Kikuchi J., Yogo K., Ogawa T., Takeya T. (2007). Org. Biomol. Chem..

[cit11] Ikeda A., Hino S., Mae T., Tsuchiya Y., Sugikawa K., Tsukamoto M., Yasuhara K., Shigeto H., Funabashi H., Kuroda A., Akiyama M. (2015). RSC Adv..

[cit12] Ikeda A., Ashizawa K., Tsuchiya Y., Ueda M., Sugikawa K. (2016). RSC Adv..

[cit13] Culver H. R., Clegg J. R., Peppas N. A. (2017). Acc. Chem. Res..

[cit14] Dimatteo R., Darling N. J., Segura T. (2018). Adv. Drug Delivery Rev..

[cit15] Vázquez-González M., Willner I. (2020). Angew. Chem., Int. Ed..

[cit16] Rao Z., Inoue M., Matsuda M., Taguchi T. (2011). Colloids Surf., B.

[cit17] Rao Z., Taguchi T. (2011). Polym. Degrad. Stab..

[cit18] Rodal S. K., Skretting G., Garred O., Vilhardt F., van Deurs B., Sandvig K. (1999). Mol. Biol. Cell.

[cit19] Castagne D., Dive G., Evrard B., Frédérich M., Piel G. (2010). J. Pharm. Pharm. Sci..

[cit20] Szejtli J., Cserhati T., Szogyi M. (1986). Carbohydr. Polym..

[cit21] Nishijo J., Mizuno H. (1998). Chem. Pharm. Bull..

[cit22] Piel G., Piette M., Barillaro V., Castagne D., Evrard B., Delattre L. (2007). Int. J. Pharm..

[cit23] Ikeda A., Iwata N., Hino S., Mae T., Tsuchiya Y., Sugikawa K., Hirao T., Haino T., Ohara K., Yamaguchi K. (2015). RSC Adv..

[cit24] Ikeda A., Funada R., Sugikawa K. (2016). Org. Biomol. Chem..

[cit25] Goto Y., Hino S., Sugikawa K., Kawasaki R., Ikeda A. (2020). Asian J. Org. Chem..

[cit26] Tamesue S., Takashima Y., Yamaguchi H., Shinkai S., Harada A. (2010). Angew. Chem., Int. Ed..

[cit27] Tamura A., Yui N. (2018). J. Controlled Release.

[cit28] Kiss T., Fenvyesi F., Bacskay I., Varadi J., Fenyvesi E., Ivanvi R., Szente L., Tsaki A., Vecsemyes M. (2010). Eur. J. Pharm. Sci..

[cit29] Merkus F. W. H. M., Schipper N. G. M., Verhoef J. C. (1996). J. Controlled Release.

[cit30] Gould S., Scott R. (2005). Food Chem. Toxicol..

[cit31] Uekama K., Fujinaga T., Hirayama F., Otagiri M., Yamasaki M. (1982). Int. J. Pharm..

[cit32] Furune T., Ikuta N., Ishida Y., Okamoto H., Nakata D., Terao K., Sakamoto N. (2014). Beilstein J. Org. Chem..

[cit33] French D., Levine M. L., Pazur J. H., Norberg E. (1949). J. Am. Chem. Soc..

[cit34] Okada M., Kamachi M., Harada A. (1999). J. Phys. Chem. B.

[cit35] Kirby C., Clarke J., Gregoriadis G. (1980). Biochem. J..

[cit36] Piel G., Piette M., Barillaro V., Castagne D., Evrard B., Delattre L. (2007). Int. J. Pharm..

[cit37] Allen T. M., Cullis P. R. (2013). Adv. Drug Delivery Rev..

[cit38] Ohtani Y., Irie T., Uekama K., Fukunaga K., Pitha J. (1989). Eur. J. Biochem..

