# Supplementary material for: Cyclodextrin-induced release of drug-entrapping liposomes associated with the solation of liposome gels
Source: RSC Adv. 2022 Aug 10;12(34):22202–9. doi: 10.1039/d2ra03837d (PMC9364157; doi:10.1039/d2ra03837d)
Supplement: RA-012-D2RA03837D-s001 [file RA-012-D2RA03837D-s001.pdf]

Electronic Supplementary Material (ESI) for RSC Advances.
This journal is © The Royal Society of Chemistry 2022

Electronic Supplementary Information

Cyclodextrin-induced release of drug-entrapping liposomes associated with the solation of liposome gels

Hiromu Yamada,[a] Keita Yamana,[a] Riku Kawasaki,[a] Kazuma Yasuhara[b] and Atsushi Ikeda *[a]

[a]Applied Chemistry Program, Graduate School of Advanced Science and Engineering, Hiroshima University, 1-4-1 Kagamiyama, Higashi-Hiroshima 739-8527, Japan.

[b]Division of Materials Science, Graduate School of Science and Technology and Center for Digital Green-innovation, Nara Institute of Science and Technology, 8916-5 Takayama-cho, Ikoma, Nara 630-0192, Japan.

Table S1. Hydrodynamic diameters (D_{hy}) of sols[a] after the addition of α-CDx determined by dynamic light scattering at 50 °C.

CDx	**1**/% (w/v)	[CDx]	[DPPC]	D_{hy}/nm	PDI[b]
α-CDx	0	7.5	2.5	93.6 ± 2.2	0.09
α-CDx	0	75	2.5	1038 ± 357	0.80

[a]Sol solutions were diluted to 1/10 using water. The final concentrations of CDx and DPPC were 0.75 and 0.25 mM, respectively. [b]PDI: Polydispersity index.

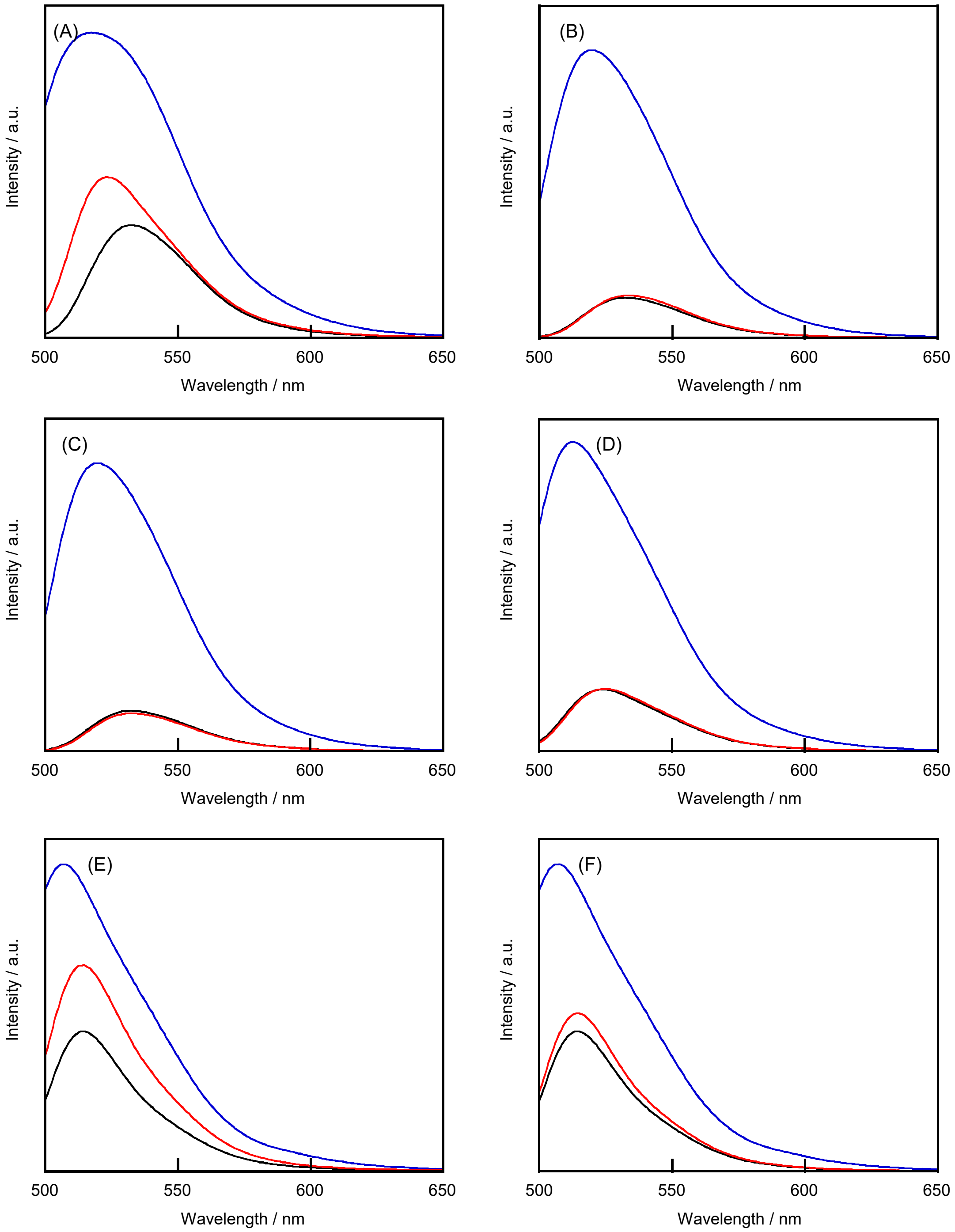

Fig. S1 Fluorescence spectra of the DPPC liposome encapsulating calcein without **1** (50 μL) after the addition of water (50 μL) (black) or a 15 mM aqueous solution (50 μL) (red) of (A) β-CDx, (B) DMe-β-CDx, (C) TMe-β-CDx, and (D) γ-CDx and a 50 mM aqueous solution (50 μL) of (E) DMe-β-CDx and (F) γ-CDx, and after the addition of 222 mM Triton X-100 (10μL) (blue). The final concentrations of the mixtures (1.2 mL) were [DPPC] = 2.5 mM and [CDx] = 0, 7.5, or 75 mM. Because the solutions of (D), (E), and (F) were cloudy after the addition of Triton X-100 in the case of (D) and (F) or after the addition of DMe-β-CDx in the case of (E), all solutions of (D), (E), and (F) were diluted to 1/2, 1/10, and 1/10, respectively, using water.

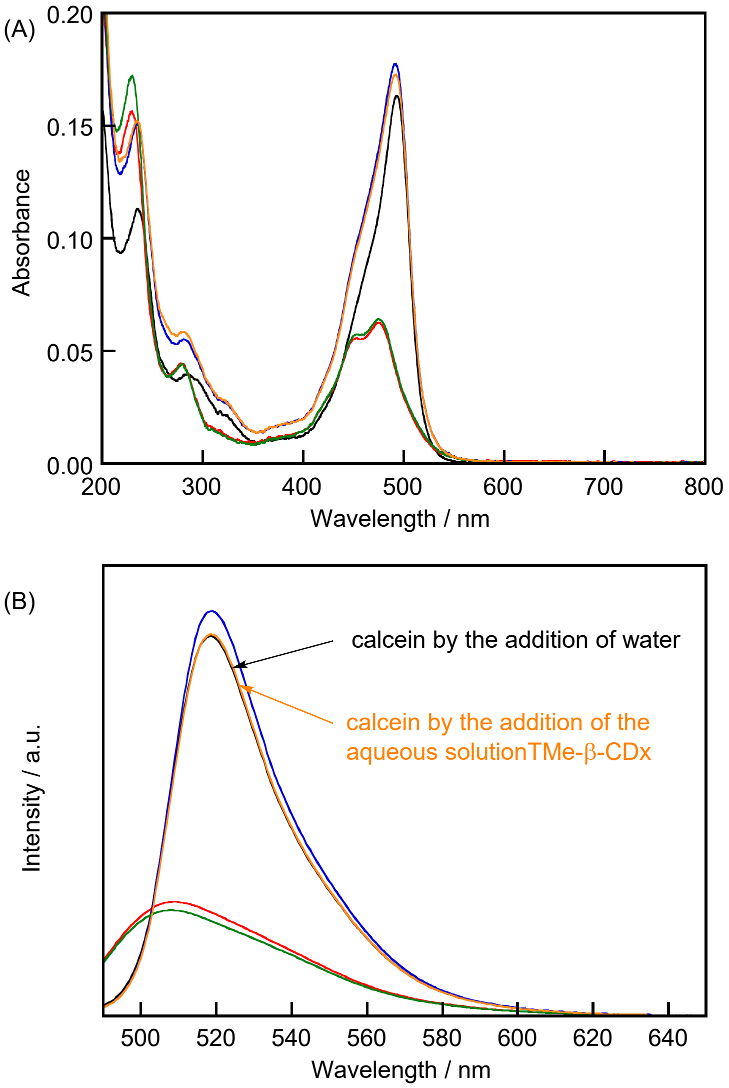

Fig. S2 (A) UV–Vis absorption and (B) fluorescence (excitation wavelength of 475 nm) spectra of the aqueous solutions of calcein by the addition of water (black) and the aqueous solutions of β-CDx (red), DMe-β-CDx (blue), TMe-β-CDx (orange), and γ-CDx (green) using 1-mm and 1-cm cells for UV–Vis absorption and fluorescence spectra, respectively, 20°C, [calcein] = 0.038 mM, [CDx] = 0.75 mM).

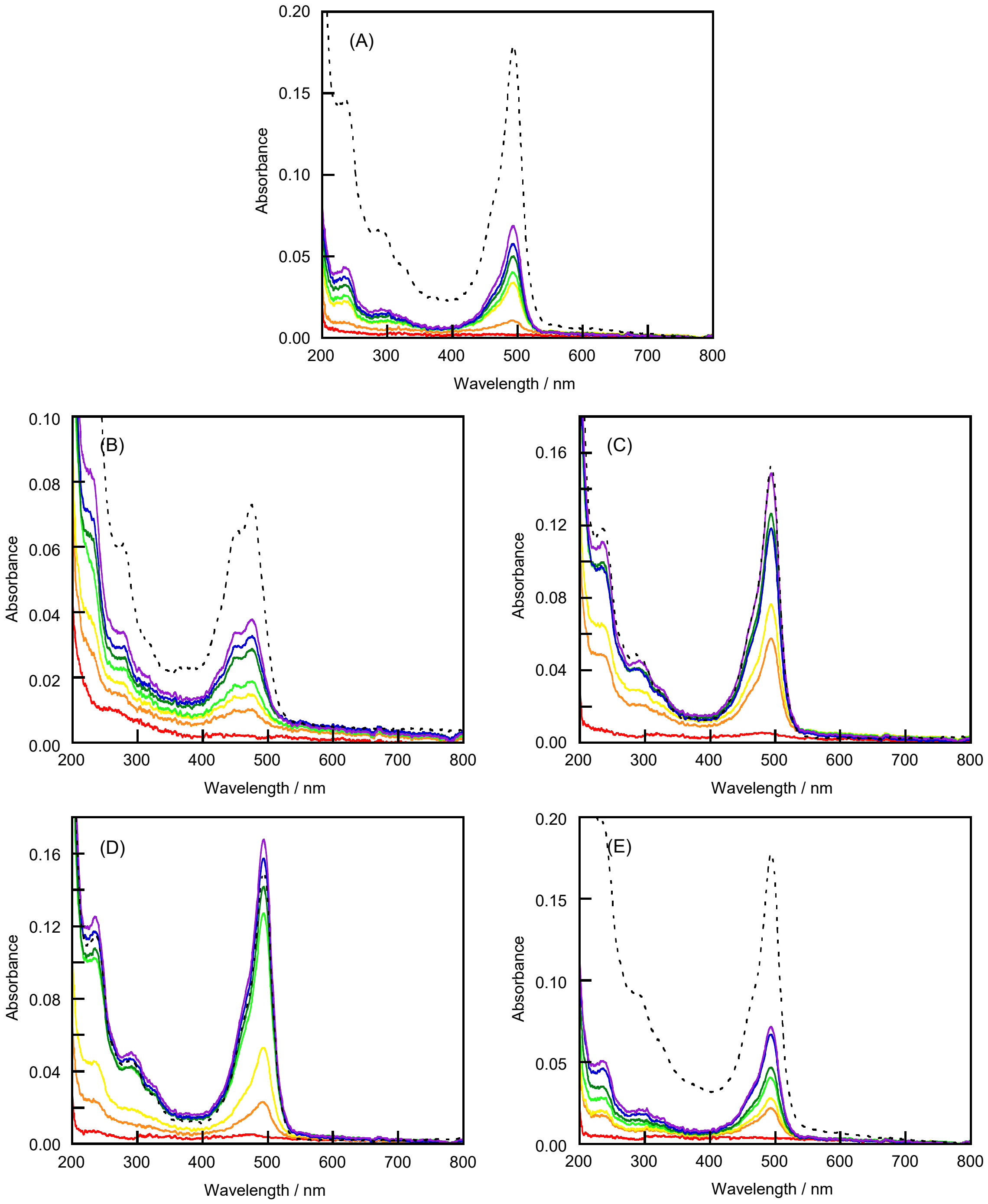

Fig. S3 UV–Vis absorption spectra of calcein in the bulk solution after the addition of (A) water, and the aqueous solutions of (B) β-CDx, (C) DMe-β-CDx, (D) TMe-β-CDx, and (E) γ-CDx kept at ambient temperature with incubation times of 0 (red), 10 (orange), 20 (yellow), 30 (light green), 40 (green), 50 (blue), and 60 (purple) min using a cell path length of 1 mm. The black dotted lines show calcein (5 mM) in the absence of (A) CDx and in the presence of (B) β-CDx, (C) DMe-β-CDx, (D) TMe-β-CDx, and (E) γ-CDx. The final concentrations of the mixtures (1.2 mL) were [DPPC] = 2.5 mM, **1**: 1.5% (w/v), and [CDx] = 0 or 7.5 mM.

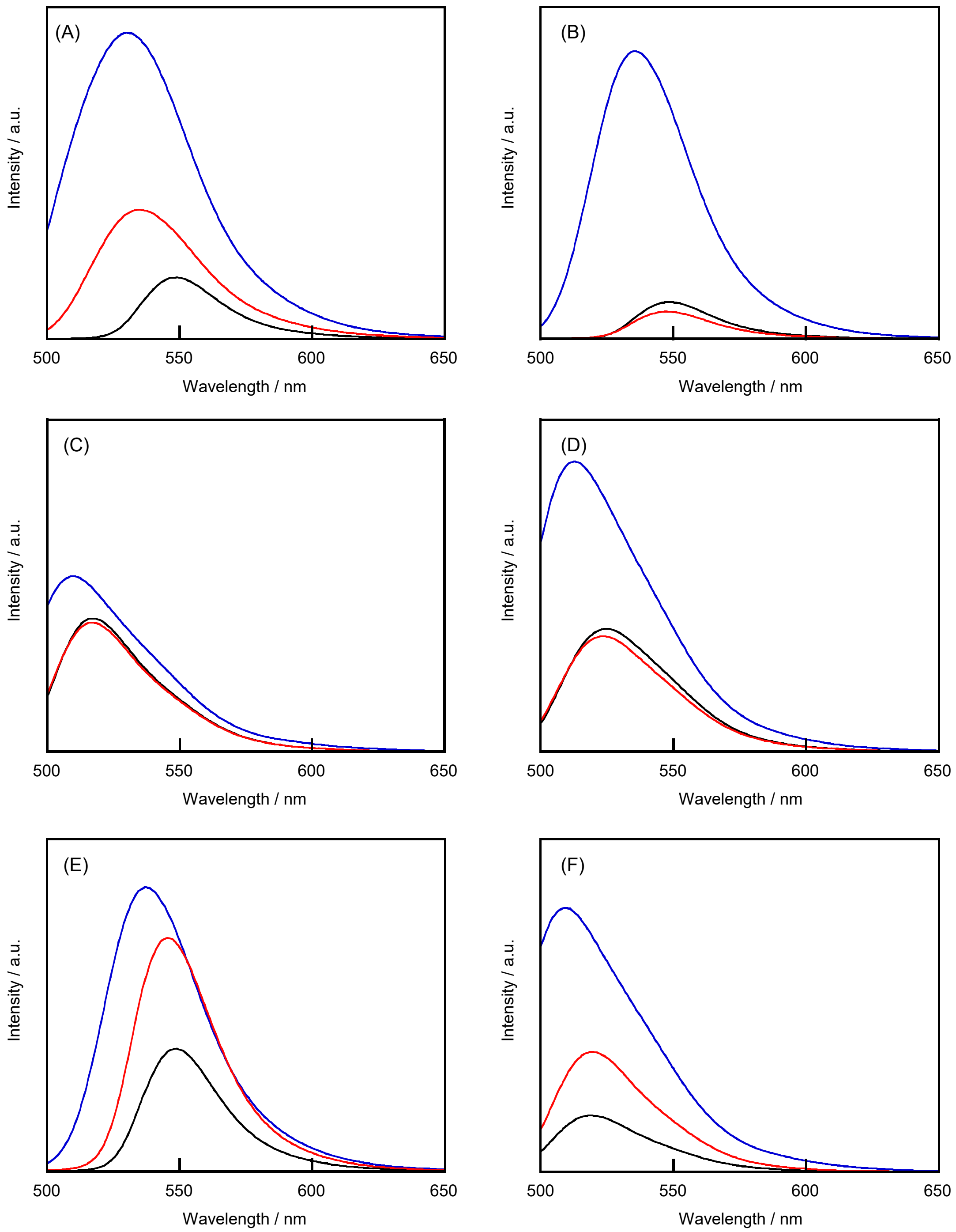

Fig. S4 Fluorescence spectra of the DPPC liposome encapsulating calcein with **1** (600 μL) after the addition of water (600 μL) (black) or a 15 mM aqueous solution (600 μL) (red) of (A) β-CDx, (B) DMe-β-CDx, (C) TMe-β-CDx, and (D) γ-CDx and a 150 mM aqueous solution (600 μL) of (E) DMe-β-CDx and (F) γ-CDx, and after the addition of 222 mM Triton X-100 (120 μL) (blue). The final concentrations of the mixtures (1.2 mL) were [DPPC] = 2.5 mM and [CDx] = 0, 7.5, or 75 mM. Because the interaction between calcein and TMe-β-CDx decreased the fluorescence intensity, all solutions in (C) were diluted to 1/2 using water. Furthermore, because the solutions in (F) were cloudy after the addition of Triton X-100, all solutions in (F) were diluted to 1/10 using water.

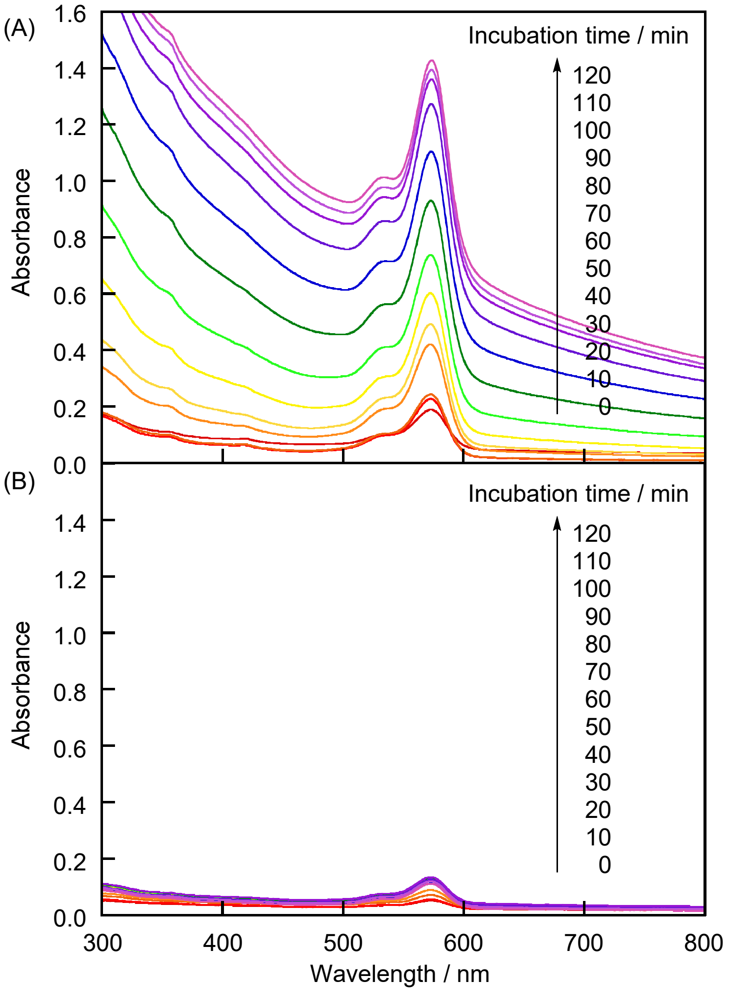

Fig. S5 UV–Vis absorption spectra of (A) an aqueous solution of TMe-β-CDx and (B) saline in the presence of liposome gels containing **3** at the bottom of the UV-cell at incubation times of 0, 10, 20, 30, 40, 50, 60, 70, 80, 90, 100, 110, and 120 min at 25°C ([DPPC] = 2.5 mM, **1**: 1.5%(w/v), [**3**] = 25 μM, (A) [TMe-β-CDx] = 7.5 mM, (B) [TMe-β-CDx] = 0 mM).

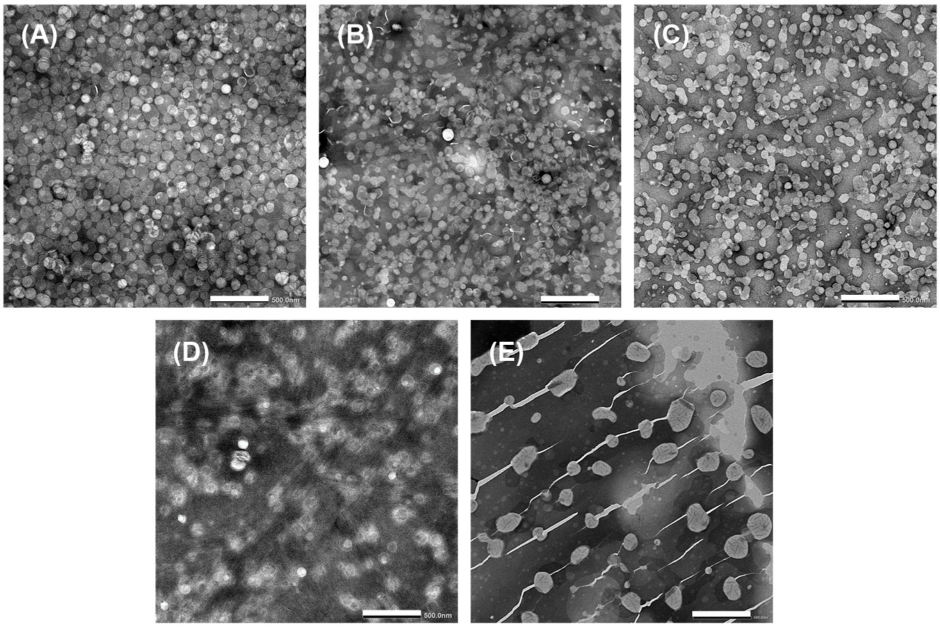

Fig. S6 TEM images of the sol solution obtained from the liposome gel (600 μL) after the addition of 15 mM aqueous solutions (600 μL) of (A) β-CDx, (B) DMe-β-CDx, (C) TMe-β-CDx, and (D) γ-CDx and 150 mM aqueous solutions (600 μL) of (E) DMe-β-CDx and (F) γ-CDx. The scale bars correspond to 500 nm.

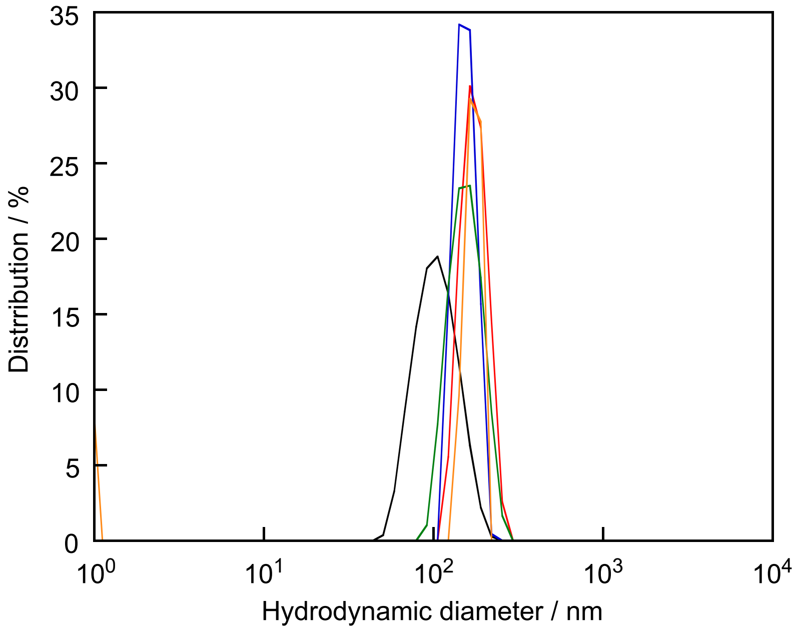

Figure S7. DLS size distribution profile for liposomes (black) and the sol solution obtained from the liposome gel (600 μL) after the addition of 15 mM aqueous solutions (600 μL) of β-CDx (red), DMe-β-CDx (blue), TMe-β-CDx (green), and γ-CDx (orange).

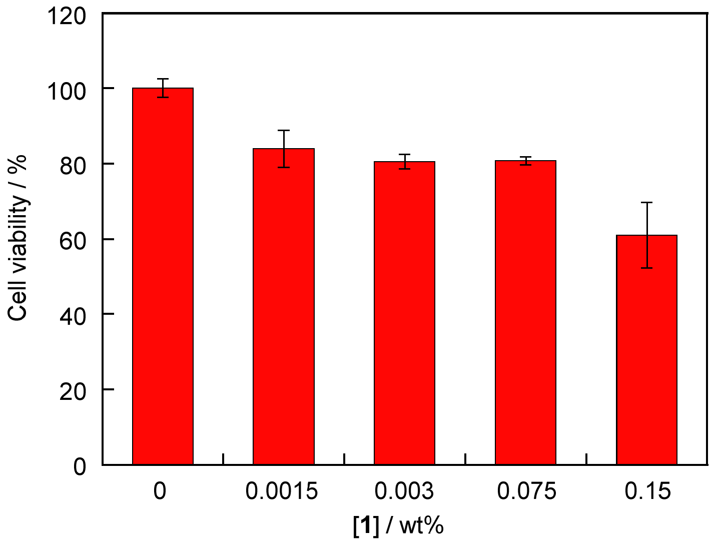

Fig. S8 Viability of Colon26 cells treated with various concentrations of **1**. Cell viability was evaluated 24 h after treatment as described in the Experimental Section. Cell viability data were confirmed implementing the CCK-8 method. Error bars represent the values for the standard deviation for n = 3.
